# Clinical characteristics of patients with epiretinal membrane-Foveoschisis

**DOI:** 10.1007/s00417-022-05940-y

**Published:** 2022-12-28

**Authors:** Annegret Hetzel, Jonas Neubauer, Faik Gelisken

**Affiliations:** Department of Ophthalmology, Eberhard-Karls-University, Elfriede-Aulhorn-Str. 7, 72076 Tuebingen, Germany

**Keywords:** Degenerative lamellar macular hole, Epiretinal membrane, Foveoschisis, Lamellar macular hole, Optical coherence tomography, Tractional lamellar hole

## Abstract

**Purpose:**

The purpose of this study is to investigate the clinical and morphological characteristics of epiretinal membrane (ERM)-Foveoschisis.

**Methods:**

Medical charts of 2088 patients diagnosed with idiopathic ERM were screened and eyes with ERM-Foveoschisis were included. All eyes underwent a complete ophthalmological examination including spectral domain optical coherence tomography (SD-OCT). OCT features and best corrected visual acuity (BCVA) were analysed. ERM-Foveoschisis was defined as *open, closed, elevated* or *flat* based on the OCT features. Ellipsoidal zone (EZ) abnormality, intraretinal cystoid spaces, central foveal thickness (CFT), posterior vitreous detachment (PVD) and lens status were assessed.

**Results:**

One hundred-sixty-six patients (175 eyes) (72% female, mean age 70.46 years) were included. Incidence of ERM-Foveoschisis was 6.7%. *Open* type was seen in 86.8% and had a significantly better mean BCVA than *closed* type (*p* = 0.01). No statistically significant difference of mean BCVA was noted between the *elevated* and *flat* types. Mean BCVA was significantly lower in eyes with EZ abnormality (*p* = 0.03) and eyes with intraretinal cystoid spaces (*p* = 0.02). Patients with ‘closed’ ERM-Foveoschisis showed a significant higher median CFT than ‘open’ ERM-Foveoschisis (respectively, 364 µm and 176 µm, *p* < 0.001). A total of 81.9% eyes had PVD.

**Conclusion:**

We differentiated four morphological types of ERM-Foveoschisis based on the OCT examination. *Closed* ERM-Foveoschisis presented with a higher CFT and lower BCVA than the *open* type. ERM-Foveoschisis with cystoid intraretinal spaces presented with a lower BCVA. The impact of the morphological types of the ERM-Foveoschisis on the clinical course and for therapy decision requires further long-term studies.


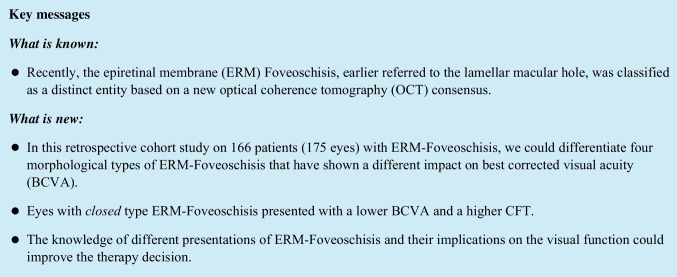


## Introduction

Lamellar macular hole (LMH) has been first described on the basis of the slit lamp biomicroscopy by Gass in 1975 [1] as the partial thickness retinal defect of the central retina. In the last years, the spectral-domain optical coherence tomography (SD-OCT) has improved the visualization of the macular microstructure [2–4] and became the gold standard for the analysis of vitreomacular interface diseases [5, 6]. Following the description of OCT characteristics of LMH by Gaudric et al. [7], further classifications were made [8–10]. Two different types, the tractional and the degenerative LMH, were proposed by Govetto et al. [9].

Recently, a new OCT-based classification for LMH, pseudo macular holes (PMH) and idiopathic epiretinal membrane-Foveoschisis (ERM-Foveoschisis) was proposed by an international panel of vitreoretinal experts [11]. The ERM-Foveoschisis, earlier referred to the LMH or to tractional LMH, was classified as a distinct entity apart from the LMH. In contrast to the LMH, the neurosensory tissue loss is not a diagnostic feature in ERM-Foveoschisis [11].

Even though there are established diagnostic OCT criteria, limited clinical knowledge exists for the ERM-Foveoschisis, since these eyes were analysed formerly within the LMH, composed of “degenerative” and “tractional” type of LMH.

The aim of this study was to investigate the clinical and morphological characteristics of a large group of patients diagnosed with ERM-Foveoschisis.

## Methods

This retrospective, monocentre, observational study was approved by the ethical committee (Reference number: 177/2020BO2) of Eberhard-Karls-University Tübingen and adhered to the declarations of Helsinki.

The electronic patient charts of 2088 consecutive patients with the diagnosis of ERM between January 2008 and January 2016 were screened retrospectively by a retinal specialist (FG). Patients with idiopathic ERM and Foveoschisis identified on the OCT examination [11] were included into the analysis. Exclusion criteria were (1) poor quality of OCT images, (2) secondary ERM (intraocular inflammation, retinal hole, retinal laser photocoagulation, cryosurgery), (3) past ocular trauma, (4) high myopia (< -6 dioptres), (5) dome-shaped maculopathy, (6) myopic traction maculopathy, (7) previous intraocular surgery except the cataract surgery, (8) coexistence of ocular diseases that may have an impact on the macular morphology and the BCVA (vascular retinal diseases, intermediate or advanced age-related macular degeneration, diabetic maculopathy, advanced glaucoma, amblyopia, retinal dystrophies), (9) LMH (formerly “degenerative” type), and (10) vitreofoveal traction without ERM.

All patients had a complete ophthalmologic examination including an eye-tracked SD-OCT (Heidelberg Engineering GmbH, Heidelberg, Germany) of the macula. The OCT examination protocol included a fovea centred 20 × 15-degree volume scan with 19 B-scans and a two-radial high-resolution scan through the fovea.

Elevation of the posterior hyaloid was noted on the fovea-centred OCT scans. The visibility of the Weiss Ring and video records in eyes, which were operated subsequently, did allow in some eyes the identification of the separation of the posterior vitreous from the optic disc.

ERM is a hyperreflective linear band on the surface of the inner retina [11]. Foveoschisis is defined as a separation of the outer nuclear layer (ONL) from the outer plexiform layer (OPL) of the retina at the level of the Henle fibre layer [11].

**Fig. 1:**
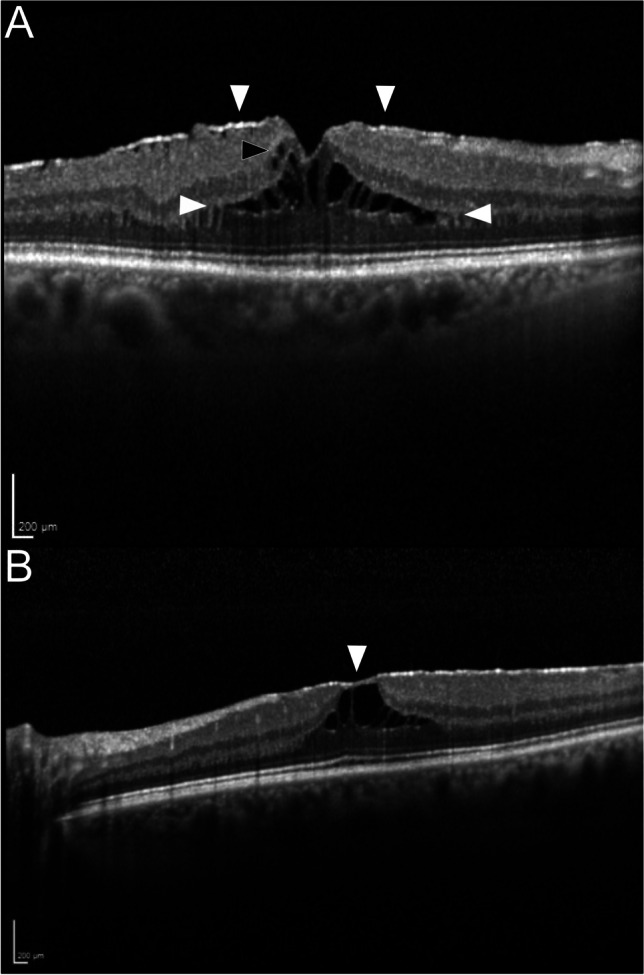
SD-OCT B-Scans of the *open* and *closed* type ERM-Foveoschisis. **a** The ERM (white arrowheads) spares the fovea in *open* type ERM-Foveoschisis. Note the separation of the outer nuclear layer from the outer plexiform layer (open white arrowheads) and the intraretinal cystoid spaces (black arrowhead). **b** The ERM covers the fovea in *closed* type ERM-Foveoschisis (arrowhead)

**Fig. 2:**
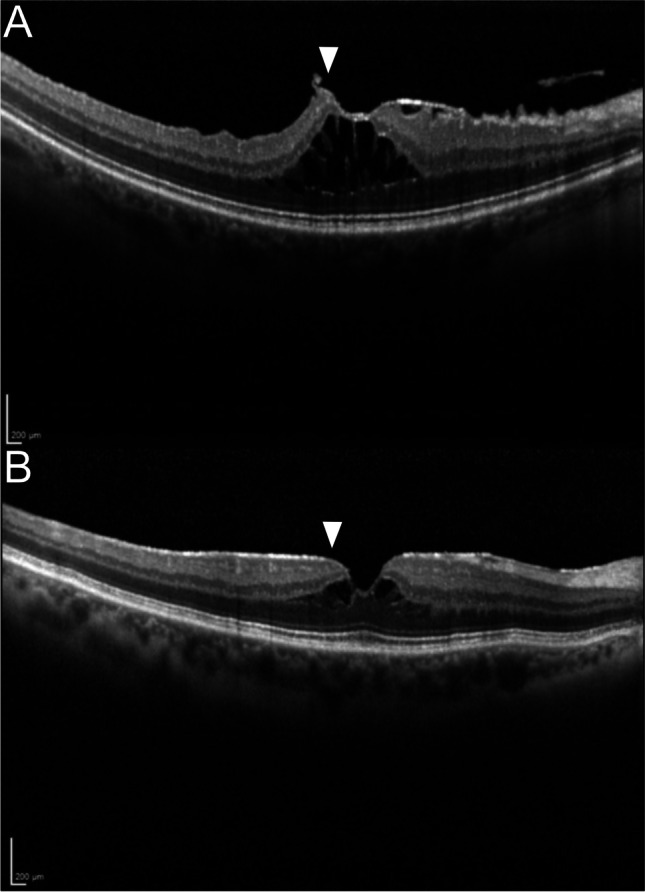
SD-OCT B-Scans of the *elevated* and *flat* type ERM-Foveoschisis. **a** The edges of the foveal border are higher than the retinal surface in *elevated* type ERM-Foveoschisis (arrowhead). **b** The edges of the foveal borders are on the same level with the rest of the foveal surface, defined as *flat* type of ERM-Foveoschisis (arrowhead)

In this study, we defined the types of ‘open and ‘closed’ ERM-Foveoschisis depending on the coverage of the ERM over the fovea. Eyes with ERM-Foveoschisis were classified as ‘open’, if the ERM spared the fovea. In turn, eyes with ERM-Foveoschisis were classified as ‘closed’, if the ERM covered the fovea completely (Fig. 1a and b). Borderline cases were classified as ‘open’ if a small discontinuation (< 100 µm) of the ERM was seen in at least two consecutive OCT scans through the fovea. If the discontinuation was not located over the fovea, the ERM-Foveoschisis was classified as ‘closed’. Eyes with ‘open’ ERM-Foveoschisis were further classified as *flat* or *elevated*, regarding the elevation of the foveal edges. ERM-Foveoschisis was defined as ‘flat’ type if the edges of the foveal borders were on the same level with the rest of the retinal surface and ‘elevated’ type if the edges of the foveal border were higher than the level of the retinal surface (Fig. 2a and b). The elevation could be found in one or more quadrants.

Other features, such as epiretinal proliferations (EP), alterations of the ellipsoid zone (EZ), fovea base irregularity and intraretinal cystoid spaces, were also noted, if present. Intraretinal cystoid spaces, as seen hyporeflective lesions on OCT, are small-sized, round or elliptical and are located in the ONL or OPL. They can be seen in ERM-Foveoschisis and may offer difficulties in differentiation from the oval-vertical oriented schitic spaces. The central foveal thickness (CFT) was measured manually with the caliper function of the Heidelberg Eye explorer between the ILM and the inner surface of the retinal pigment epithelium.

Posterior vitreous detachment (PVD) was detected on the OCT scans over the macula. We noted whether a partial, complete or no PVD was present. The OCT features were analysed by two examiners independently (AH, JN). In cases of discrepancies, a consensus was found after discussion and re-evaluation of the case with the experienced examiner (FG).

Statistical analysis was performed by using JMP version 16.0.0 (SAS Institute Inc., SAS Campus Drive, Cary, North Carolina 27,513, USA). The obtained Snellen equivalent of BCVA was converted into logMAR for statistical analysis. Qualitative measurements were presented as mean ± standard deviation (SD) or median with first and third quartile and were compared by using the Student’s *t* test or the Mann–Whitney *U* test. Quantitative measurements were presented as absolute value and percentage and were analysed by using the chi^2^ test. Spearman rank correlation was used as a measure of a monotonic association (Spearman correlation coefficient ρ). Statistical significance was assumed 5% (*p* = 0.05) for all tests.

## Results

After screening the medical charts of 2088 patients (2625 eyes) with idiopathic ERM, 166 patients (175 eyes) fulfilled the criteria for ERM-Foveoschisis and were included into the study. The incidence of ERM-Foveoschisis within the patients of idiopathic ERM was found as 6.7%.

One hundred-twenty patients (72%) were female. There was no difference in gender distribution neither in patients with ‘open’ or ‘closed’ ERM-Foveoschisis (females 74% vs. 61%, *p* = 0.2) nor in patients with ‘flat’ or ‘elevated’ types (females 72% vs. 75%, *p* = 0.6). The mean age was 70.46 years (range, 47–89 years). Patients with ‘open’ or ‘closed’ ERM-Foveoschisis did not differ in age (71.8 years (**±**6.6 years) vs. 70.2 years (**±**7.6 years), *p* = 0.15). Patients with ‘flat’ ERM-Foveoschisis were significantly older than patients with ‘elevated’ ERM-Foveoschisis (71.7 years (**±**7.7 years) vs. 69.3 years (**±**7.4 years), *p* = 0.02). Bilateralism was seen in nine patients. ‘Closed’ type ERM-Foveoschisis was predominant in bilateral affected patients: Seven patients (77.8%) presented with ‘closed’ type ERM-Foveoschisis in both eyes, and two patients (22.2%) had ‘closed’ ERM-Foveoschisis in one eye but ‘open’ ERM-Foveoschisis in the fellow eye. Patients with ‘open’ ERM-Foveoschisis presented mainly with ‘elevated’ type ERM-Foveoschisis (68.7%) than with ‘flat’ type ERM-Foveoschisis (31.3%). Ninety-four (53.7%) right eyes and 81 (46.3%) left eyes were recorded. Of the 175 included eyes, 79.4% were phakic. Baseline characteristics are summarized in the Table 1.

**Table 1:** Baseline characteristics of eyes with ERM-Foveoschisis

	Total 166 patients (175 eyes) *N* affected eyes (%)	BCVA [LogMAR]	*p* value
Laterality			
Right	94 (53.7%)		0.15^2^
Left	81 (46.3%)		
Lens			
Phakic	139 (79.4%)	0.25 ± 0.17	0.16^1^
Pseudophakic	36 (20.6%)	0.29 ± 0.19	
Types of ERM-Foveoschisis			
Closed	23 (13.1%)	0.34 ± 0.21	**0.01**^**1**^
Open	152 (86.8%)	0.24 ± 0.17	
Flat	60 (39.5%)	0.22 ± 0.21	0.13^1^
Elevated	92 (60.5%)	0.26 ± 0.18	
OCT features			
Ellipsoid zone abnormality			
Present	15 (9.9%)	0.33 ± 0.19	**0.03**^**1**^
Not present	160 (90.1%)	0.22 ± 0.16	
Epiretinal proliferations			
Present	20 (12.2%)	0.21 ± 0.15	0.22^1^
Not present	155 (87.8%)	0.26 ± 0.18	
Intraretinal cystoid spaces			
Present	92 (52.9%)	0.28 ± 0.19	**0.02**^**1**^
Not present	83 (47.1%)	0.22 ± 0.15	
fovea base irregularity			
Present	45 (26.3%)	0.25 ± 0.19	0.74^1^
Not present	130 (73.7%)	0.26 ± 0.17	

*BCVA*, best-corrected visual acuity; *ERM*, epiretinal membrane; *OCT*, Optical coherence tomography; *N*, number; *SD*, standard deviation; *p*-values with statistical significance are highlighted bold

^1^Student’s *t* test

^2^Chi^2^ test

The mean best corrected visual acuity (BCVA) was 0.25 ± 0.18 logMAR. No significant differences were seen based on the status of the lens and the BCVA. ‘Open’ type ERM-Foveoschisis had significantly better mean BCVA (0.24 ± 0.17 logMAR) in comparison to the ‘closed’ type ERM-Foveoschisis (0.34 ± 0.21 logMAR, *p* = 0.01). No statistically significant difference of mean BCVA was noted between ‘elevated’ and ‘flat’ ERM-Foveoschisis (0.26 ± 0.18 logMAR vs. 0.22 ± 0.21 logMAR, *p* = 0.13).

The mean BCVA in 15 eyes with EZ abnormality (0.33 ± 0.19 log MAR) was significantly lower than in 137 eyes without EZ abnormality (0.22 ± 0.16 logMAR, *p* = 0.03). Likely, the mean BCVA was found significantly lower in eyes with intraretinal cystoid spaces (0.28 ± 0.19 logMAR) vs. without (0.22 ± 0.15 logMAR, *p* = 0.02).

The presence of the EP and fovea base irregularity did not influence the mean BCVA statistically (Table 1).

Patients with *‘*closed’ ERM-Foveoschisis showed a significant higher median CFT than ‘open’ ERM-Foveoschisis (respectively, 364 µm (1. quartile 303 µm; 3. quartile 447 µm) and 176 µm (1. quartile 161 µm; 3. quartile 213 µm), *p* < 0.001). Median CFT did not differ between patients with ‘flat’ or ‘elevated’ ERM-Foveoschisis (175.5 µm (1. quartile 158.8 µm; 3. quartile 219.8 µm) vs. 176 µm (1. quartile 161.3 µm; 3. quartile 203.3 µm), *p* = 0.71).

In ‘closed’ ERM-Foveoschisis, lower BCVA correlated with higher CFT (ρ= 0.53, *p* = 0.001) but not in ‘open’ ERM-Foveoschisis (ρ = 0.12, *p* = 0.14). Regarding the subtypes, there was no correlation of BCVA with CFT neither in ‘flat’ type ERM-Foveoschisis (ρ= 0.15, *p* = 0.16) nor in ‘elevated’ type ERM-Foveoschisis (ρ = 0.06, *p* = 0.6).

Vitreous was completely or partially detached over the macula in 81.9% of the eyes. Complete macular vitreous detachment was present in 75.6% of the eyes. No vitreous detachment over the macula was seen in 13.1% of the eyes, and the status of the vitreous detachment in nine eyes was non-gradable (5.1%). PVD including the optic disc was seen in 58.1%.

## Discussion

In our study, we proposed an OCT-based morphological classification for the ERM-Foveoschisis. The types of ERM-Foveoschisis, i.e. *closed, open, elevated* or *flat*, were analysed in respects to the BCVA and other OCT features. *Closed* type ERM-Foveoschisis presented with a lower mean BCVA than the *open* type.

Some of the demographic and clinical characteristics of ERM-Foveoschisis in our cohort differed from those of previous reports on LMH and ERM-Foveoschisis.

One difference is the younger age of our patients with ERM-Foveoschisis than those of previously reported studies of LMH [8, 9, 12]. The cause of this discrepancy is possibly due to the different compositions of the analysed cohorts. Unlike our study, the previous LMH cohorts included beside the tractional also the degenerative type of LMH, seen mostly in elderly. Interestingly, patients of our study with ‘flat’ ERM-Foveoschisis were older than patients with ‘elevated’ ERM-Foveoschisis. Females showed dominance in our study group like in another study with a mixed cohort of tractional and degenerative LMH [12]. Some studies on ERM-Foveoschisis showed no difference in age and gender in eyes with ERM-Foveoschisis from those with LMH [13–15]. However, the last-mentioned cohorts were small and mixed with LMH or MPH. Furthermore, they did not exclude eyes with secondary ERM.

Another difference is that the prevalence of ERM-Foveoschisis within our cohort was with 6.7% higher than the prevalence of 3.1% in a cohort of 544 eyes with primary ERM reported by Lam et al. [16]. In the literature, there is little information on the prevalence of ERM-Foveoschisis, but the prevalence of ERM has been investigated widely in different populations. It varies between 2.2 and 34% [3, 17–19] depending on the ethnic group studied and on the cause of ERM. Due to the fact that ERM is a mandatory criterion for the diagnosis of ERM-Foveoschisis, it is likely that the prevalence of ERM-Foveoschisis may also vary between ethnic groups.

A further difference of clinical characteristics is the lower prevalence of intraretinal cystoid spaces within our cohort. A study by Govetto et al. showed a prevalence of intraretinal cystoid spaces of 81.4% in a series of 43 eyes with tractional LMH [9], which in turn was found in 52.9% of the 175 eyes in our study. We found a significant lower mean BCVA of 0.28 logMAR in eyes with intraretinal cystoid spaces vs. 0.22 logMAR in eyes without it. The prognostic role of the intraretinal cystoid spaces in ERM-Foveoschisis deserves further analysis.

Despite the mentioned differences, there are some concordances of clinical characteristics of ERM-Foveoschisis with previous reports.

One is the high prevalence of vitreous detachment over the macula in 81.9% within our cohort that is in line with earlier studies on patients with ERM or LMH that indicated a prevalence of PVD in 70.5–100% [12, 20–24].

Another concordance is the integrity of the EZ. Consistent with the study of Govetto et al., the eyes in our cohort showed an intact EZ in most cases. In a recent study, defects in the EZ were reported higher in eyes with LMH than in eyes with ERM-Foveoschisis resulting in a lower residual EZ and a lower BCVA [15]. Even though seen infrequently in our cohort, the EZ abnormality was associated with lower BVCA, if present.

In line with a recently published study by Nakamura et al. that reported a CFT of 184.0 µm in ERM-Foveoschisis [15], we found a median CFT of 180.5 µm, higher than previous studies of LMH that showed a mean CFT of 72–132.69 µm [8, 9, 12, 20].

In this study, the proposed morphological types of ERM-Foveoschisis have shown a different impact on BCVA and CFT. The mean BCVA of the total collective was 0.25 logMAR. ‘*Open*’ type ERM-Foveoschisis presented with a mean BCVA of 0.24 logMAR, whereas the ‘*closed*’ type ERM-Foveoschisis had a significantly lower mean BCVA of 0.34 logMAR. The ‘*elevated*’ type had a mean BCVA of 0.26 logMAR similar to the ‘*flat*’ ERM-Foveoschisis with a mean BCVA of 0.22 logMAR.

In our cohort, higher CFT was seen mostly in *closed* type ERM-Foveoschisis associated with a lower BCVA. *Open* type ERM-Foveoschisis presented with a higher BCVA and a lower CFT, but the correlation was not statistically significant. However, no correlation of BCVA or CFT was found with *flat* or *elevated* type of ERM-Foveoschisis. Our findings are in line with earlier studies that indicated a correlation of lower BCVA with higher CFT in ERM-Foveoschisis [15]. However, there are other reports of 35 eyes with ERM-Foveoschisis [14] and 43 eyes with tractional LMH [9] that indicated a lower CFT in eyes with a lower BCVA. However, the cohorts of the last-mentioned studies were smaller, and a direct comparison of these studies is limited by the fact that they did not differentiate morphological types of ERM-Foveoschisis as in our study.

The limitations of this study were based on the retrospective design. Due to the retrospective design of the study and lack of a standard questionnaire, no reliable information on the disease duration in all patients could be identified. OCT imaging was limited to the macular areas in most of the eyes. The visibility of the Weiss Ring and video records in operated eyes did allow in some eyes the identification of the separation of the posterior vitreous from the optic disc. Consequently, in some eyes, the attachment of the vitreous in respect to the optic disc could not be assessed with certainty. Autofluorescence imaging of the macula and ultrasound examination, which could provide additional information, was not performed routinely. Even though the CFT was measured in a standardized setting between the ILM and the inner surface of the retinal pigment epithelium, the manual measurement method with the caliper function of the Heidelberg Eye explorer might be a source of inaccuracy. And, as this study was cross-sectional, no evaluation of the clinical course was performed for the different morphological types of ERM-Foveoschisis.

Furthermore, to evaluate the progression from one type to another type of ERM-Foveoschisis, and to identify the OCT-biomarkers for a conversion, further long-term observations are required.

The presented study by design did not address the criteria for the therapy. In general, in ERM, macular surgery is chosen when BCVA is significantly reduced or if the patient is complaining of metamorphopsia. Documented progression of the macular pathology by OCT is also a frequent reason for surgical management in some cases. On the other hand, no standardized evidence-based surgical procedure exists for the ERM-Foveoschisis, specifically in terms of indication, timing, or surgical technique. A recent study has shown a better functional and morphological outcome in LMH with little tractional properties treated by macular surgery, including the combination of a partial ERM and ILM peeling and modified flap technique [25]. However, Figueroa et al. showed better functional and anatomical outcomes in eyes with LMH and tractional forces of the ERM that had undergone the standard vitrectomy with peeling of the ERM and ILM, compared to LMH without traction of the ERM [26]. Another study on LMH showed improved fovea morphology after macular surgery including the ILM peeling and application of highly concentrated autologous platelet-rich plasma [27].

Considering the overlapping presentations or conversions between the entities of the LMH and ERM-Foveoschisis, the use of highly concentrated autologous platelet-rich plasma may deserve further long-term studies in larger cohorts in both entities.

In summary, we differentiated four morphological presentations of the ERM-Foveoschisis. *Closed* type ERM-Foveoschisis presented with an increased CFT and lower BCVA than the *open* type. ERM-Foveoschisis with cystoid intraretinal spaces presented with a lower BCVA. The prognostic impact of the different morphological presentations of the ERM-Foveoschisis deserves further clinical research.

## Data Availability

All data relevant to the study are included in the article or uploaded as supplemental information. The manuscript or portions of it have not been previously published or presented at a meeting and are not under consideration by another journal.

## Declarations

### Disclaimer

The corresponding author attests that all listed authors meet authorship criteria and that no others meeting the criteria have been omitted.
